# Supplementary material for: Identifying subgroups with differential levels of service response to a digital screening and service navigation program for unmet social care needs
Source: PLoS One. 2026 Jan 7;21(1):e0332790. doi: 10.1371/journal.pone.0332790 (PMC12779123; doi:10.1371/journal.pone.0332790)
Supplement: Attachment — Submitted filename: Response to Reviewers.docx [file pone.0332790.s001.docx]

**Point-by-point response to Reviewers’ comments**

Dear Editor,

We thank you and the reviewers for your time in reviewing our paper and providing valuable comments that have helped to improve the current version. We have carefully considered the comments and responded to all the items. We hope the manuscript after careful revisions meet your high standards. Below we provide the point-by-point responses. All modifications in the manuscript have been highlighted as tracked changes.

Journal requirements:

When submitting your revision, we need you to address these additional requirements.

1. Please ensure that your manuscript meets PLOS ONE's style requirements, including those for file naming. The PLOS ONE style templates can be found at

<https://journals.plos.org/plosone/s/file?id=wjVg/PLOSOne_formatting_sample_main_body.pdf> and

[https://journals.plos.org/plosone/s/file?id=ba62/PLOSOne_formatting_sample_title_authors_affiliations.pdf](https://track.editorialmanager.com/CL0/https:%2F%2Fjournals.plos.org%2Fplosone%2Fs%2Ffile%3Fid=ba62%2FPLOSOne_formatting_sample_title_authors_affiliations.pdf/1/010f019a9efed5ab-09b37ce7-3658-47c7-81e8-c27ab6b59cf8-000000/AQ1Jp3ntEp1p6sZz6AichRQaLlAPlIzs6g8OwuyPjMI=238)

***Author response:*** We have carefully formatted the manuscript as per PLOS ONE's style requirements.

2. In the online submission form, you indicated that [The datasets generated and/or analysed during the current study are not publicly available due to ethical and legal reasons, including participant anonymity and privacy, but may be available from the corresponding author on reasonable request.].

All PLOS journals now require all data underlying the findings described in their manuscript to be freely available to other researchers, either 1. In a public repository, 2. Within the manuscript itself, or 3. Uploaded as supplementary information.

This policy applies to all data except where public deposition would breach compliance with the protocol approved by your research ethics board. If your data cannot be made publicly available for ethical or legal reasons (e.g., public availability would compromise patient privacy), please explain your reasons on resubmission and your exemption request will be escalated for approval.

***Author response:*** We have revised the data sharing statement in the manuscript (See Page 25, Lines 390-396).

***Modifications: “Data sharing statement:*** *The individual level data collected and analysed during the current study are not publicly available due to ethical and privacy reasons. Data sharing has been restricted by the South Western Sydney Local Health District Human Research Ethics Committee. Requests will be reviewed in accordance with the approval of the South Western Sydney Local Health District Human Research Ethics Committee (swslhd-ethics@health.nsw.gov.au) to ensure that all ethical and legal requirements for data protection are met.”*

3. Your ethics statement should only appear in the Methods section of your manuscript. If your ethics statement is written in any section besides the Methods, please move it to the Methods section and delete it from any other section. Please ensure that your ethics statement is included in your manuscript, as the ethics statement entered into the online submission form will not be published alongside your manuscript.

***Author response:*** *We confirm that the ethics statement only appears in the Methods section of your manuscript.*

4. If the reviewer comments include a recommendation to cite specific previously published works, please review and evaluate these publications to determine whether they are relevant and should be cited. There is no requirement to cite these works unless the editor has indicated otherwise.

***Author response:*** We thank the Editorial team for this information.

**Reviewer #1**

1. Overall: The manuscript provides an analysis of data collected in context of larger RCT in which they use LCA methods to identify characteristics associated with social risks and with response to digital intervention. Rural, poor, mentally ill, non-CALD populations were more likely to have social risks (by definition, since they're poor and otherwise marginalized) and trended towards being less responsive to digital intervention. No big surprises there. I would suggest authors do a little revising to abstract to clarify what they are doing, strengthen intro to contextualize work, and add to Discussion to think more deeply about what it means that largely poor, rural, mentally ill, but NONCALD people seemed to do worse. They don't really engage with the finding around race/language and in fact seem to suggest later in discussion that more linguistic support is necessary, which feels inconsistent with findings. Additional notes that I took while reading are included below.

***Author response:*** We thank the reviewer for this feedback. We have revised the manuscript in several key areas. First, we have clarified the study aims in the Abstract to better reflect our analytical focus and intended contribution (See Page 3, Lines 49-52). Second, we have strengthened the Introduction to provide clearer context around the populations most affected by unmet social care needs and the importance of examining differential responses to digital service navigation (See Page 6, Lines 102-112). Third, we have expanded the Discussion to engage more deeply with the finding that participants who were predominantly from low socioeconomic status, rural, and experiencing mental health difficulties but not from CALD backgrounds had poorer outcomes (See Pages 20-21, Lines 312-327).

Abstract

1. Opening sentence is: "Digital tools are increasingly integrated into health systems to identify and support needs, yet their effectiveness remains unclear among priority communities.” The relationship between digital tools and needs is unclear. Whose needs? Effectiveness for what?

***Author response:*** We agree that the opening sentence in the abstract is ambiguous. We have revised the text to explicitly state that we are referring to the unmet social care needs of families and the effectiveness of these tools in addressing those needs (See Page 3, Lines 49-51).

***Modifications:*** *“Digital screening and navigation interventions are increasingly integrated into health systems to identify and support* ***families’ unmet social care needs****, yet their effectiveness in* ***improving outcomes*** *remains unclear among priority communities.”*

2. Second sentence transitions to “digital intervention” but what relationship does that have with digital tools? Just needs clearer link to what they’re studying in this article.

***Author response:*** We have now refined the first sentence to start with “Digital screening and navigation interventions” to better link with the second sentence and ensure consistency (See Page 3, Lines 49-52).

***Modifications:*** *“****Digital screening and navigation interventions*** *are increasingly integrated into health systems to identify and support families’ unmet social care needs, yet their effectiveness in improving outcomes remains unclear among priority communities. We hypothesise that responses to* ***such digital interventions*** *might vary based on sociodemographic and psychosocial characteristics.”*

3. Somewhere in abstract it would be helpful to explain that authors are looking at patient, caregiver/household needs. For instance, in methods section of abstract, it would help orient reader if the word “patient” or “household” were inserted before “clinical and sociodemographic” in this sentence: "Latent class analysis was used to identify subgroups based on clinical and sociodemographic factors."

***Author response:*** We have revised the sentence and added “subgroup of families” and based on “parental and child” clinical and sociodemographic factors for clarity (See Page 3, Lines 56-57).

***Modifications:*** *“Latent class analysis was used to identify subgroups of* ***families*** *based on* ***parental and child*** *clinical and sociodemographic factors.”*

Introduction

1. They indicate intervention has been evaluated in RCT, but don’t clearly share findings from the evaluation unless that’s the references 8 and 10? The structure of the first five sentences of that paragraph is really confusing to a naive reader.

***Author response:*** We thank the reviewer for this feedback. We have rewritten the first five sentences of the paragraph in the introduction to clarify the findings from previous evaluations of the WMG-E tool. Specifically, we now explicitly state that prior evaluations have demonstrated the feasibility, acceptability, and ability of the WMG-E tool to increase service connections among families from priority populations (See Pages 5-6, Lines 93-102).

***Modifications:*** *“Watch Me Grow-Electronic (WMG-E) is a digital screening and navigation program that was developed to identify and support child developmental concerns alongside parental mental health and unmet social care needs among families with children aged 0-5 years [8, 9]. Previous evaluations of the WMG-E tool have demonstrated feasibility and acceptability among families from priority populations [8, 10, 11].”*

2. Then the next half of that paragraph is about what is unknown. Do the authors really mean to say that it is unclear in literature how parental education is related to unmet needs?

***Author response:*** We appreciate the reviewer’s comment and have clarified this point. We do not intend to suggest that the literature is unclear about the general relationship between parental education and unmet needs. Rather, we now explicitly state that it is unclear how parental education, alongside other sociodemographic and clinical characteristics, influences families’ engagement with the WMG-E tool and the extent to which the intervention addresses unmet social care needs (See Page 6, Lines 102-112).

***Modifications:*** *“However, less is known about how different subgroups of families within these populations respond to the intervention. In particular, it remains unclear whether certain key sociodemographic characteristics (such as child and maternal age, child’s gender, parental education level, and cultural background) and clinical profiles (including parental mental health and child developmental vulnerabilities) influence engagement with the WMG-E tool or the extent to which unmet social care needs are identified and supported via the use of the tool. Furthermore, there is a limited understanding of how unmet social needs, such as housing instability, unemployment, financial stress, transportation difficulties, and food insecurity, interact with these factors to shape differential responses to digital screening and navigation support.”*

Discussion

1. Opening of summary of findings suggests that class shaped needs and response to intervention. I don’t think that is accurate. Class was associated with needs; using language suggesting causality here is not quite right.

***Author response:*** We agree that causal language is inappropriate here. We have modified the text to reflect association rather than causation (See Page 19, Lines 266-268).

***Modifications:*** *“This study identified three distinct latent subgroups of parents based on their sociodemographic and psychosocial characteristics, which were associated with differences in social care needs and responses to the WMG-E tool.”*

2. At end of that section, they suggest, "This also highlights the critical need for tailored interventions, particularly digital models of care, to better support the challenges and capacities of different family groups.” Once I read rest of discussion, I understood (and agreed) with their point, but this sentence is a little ambiguous. In fact, because they did not test digital models vs other model in this study, they don’t know if non digital models “particularly” need tailoring.

***Author response:*** We thank the reviewer for this feedback. We agree that the original wording could be misinterpreted as implying that digital models require more tailoring than other non-digital models. We have revised the sentence to remove this implication and ensure it accurately reflects the study findings without suggesting comparisons between digital and non-digital models which was not the focus of this study (See Page 19, Lines 276-277).

***Modifications:*** *“This also highlights the critical need for tailored interventions to better support the challenges and capacities of different family groups.”*

3. Also wondering if authors can explain why they are suggesting more linguistic support (they refer to language barriers, e.g.) necessary when Class 2 is largely non CALD. I agree with suggestion, but not c/w findings, right? Or maybe I'm misunderstanding. Also maybe could describe a little more about rural/urban context in Australia and how they make sense of that.

***Author response:*** We agree with the feedback. We have revised the discussion to highlight the multiple factors contributing to the vulnerabilities of Class 2 families, including socioeconomic disadvantage, rural/remote location, limited access to services, and psychological distress. This clarifies the barriers faced by this subgroup and better contextualises their persistent unmet needs (See Pages 20-21, Lines 312-327).

***Modifications:*** *“The increase in reported social care needs among Class 2 participants reflects the concept of ‘diagnostic overload’, where screening identifies issues but fails to provide effective pathways to resolution, posing a risk of increased inequity [25]. Families in this group may experience multiple, intersecting barriers such as limited financial resources, reduced local service availability, transportation challenges, and difficulties balancing work or caregiving responsibilities which can exacerbate the challenges of accessing recommended supports. These systemic barriers are particularly salient in rural or regional settings, where services are often less accessible, waitlists are longer, and specialist supports may be scarce. This interpretation is supported by the qualitative findings from the broader trial, where families reported challenges in following through with digital referrals without hands-on support [26]. Moreover, the study was conducted during the COVID-19 pandemic associated social restrictions and health service shutdowns likely exacerbated difficulties in accessing the recommended supports. For families with low social capital and limited digital literacy, these factors may have further hindered their ability to engage with the intervention and receive timely support.”*

**Reviewer #2**

As the statistical reviewer I will focus on methods and reporting

Major
1) how did you deal with missing data? were all data complete? please state so if yes. if not, why weren't multiple imputation approaches considered?

***Author response:*** We thank the reviewer for this comment. All data were complete except for one variable (current service use), which had only three missing cases. Given the extremely small amount of missing data, multiple imputation was not necessary, and analyses were conducted using the available data.

***Modifications:*** *No changes made.*

2) the authors said "final model was selected based on the optimal balance of fit indices and interpretability". perhaps some more information is needed here for clarity, and to make analyses replicable. Specify which fit indices were prioritised (AIC, LMR-LRT other), and how interpretability was assessed. Was any sensitivity analysis conducted to assess robustness of the chosen model?"

***Author response:*** We have now elaborated on the model selection process in the Methods section (See Page 9, Lines 176-181).

***Modifications:*** *“The final model was selected based on prioritising lower AIC and BIC values, significant Lo-Mendell-Rubin likelihood ratio tests, and entropy values ≥0.70. Interpretability was assessed by examining whether classes represented conceptually meaningful and distinct clinical and sociodemographic profiles. Sensitivity analyses involved reviewing other class solutions to ensure the selected model remained robust across specifications.”*

Minor
1) what is regression for count data? Poisson? negative binomial regression? can the authors be clearer here? made clearer later, but you don't have to report things twice, the first time being unclear

***Author response:*** We have clarified the statistical method earlier in the Data Analysis section to avoid ambiguity (See Page 9, Lines 183-184).

***Modifications:*** *“Following class assignment, a zero-inflated negative binomial regression (ZINB) model with clustering to account for repeated measures at the individual level, was used to assess changes in social care needs (measured by WE CARE scores) across three time-points (baseline, 6 months, and 12 months).”*

2) "...and their interactions". can you be clearer on the interactions included in the model? it becomes a bit clearer later i think, trial arm by latent class

***Author response:*** We have explicitly listed the interaction terms in the Methods section (See Pages 9-10, Lines 188-191).

***Modifications:*** *“Interaction terms included all relevant two-way interactions (class × group, class × time, group × time) as well as the three-way interaction (class × group × time) to examine whether intervention effects differed across latent classes over time.”*

3) How was clustering accounted for? using random effects or robust standard errors? clarify how repeated measures were handled as well.

***Author response:*** We have specified that random intercepts were used to account for clustering within individuals (See Page 9, Lines 186-187).

***Modifications:*** *“Clustering was handled using random intercepts for individuals to account for within-person correlations over time.”*
