# Supplementary material for: Identifying subgroups with differential levels of service response to a digital screening and service navigation program for unmet social care needs
Source: PLoS One. 2026 Jan 7;21(1):e0332790. doi: 10.1371/journal.pone.0332790 (PMC12779123; doi:10.1371/journal.pone.0332790)
Supplement: Attachment — Submitted filename: Response_to_Reviewers_auresp_2.docx [file pone.0332790.s002.docx]

**Point-by-point response to Reviewers’ comments**

Dear Editor,

We thank you and the reviewers for your time in reviewing our paper and providing valuable comments that have helped to improve the current version. We have carefully considered the comments and responded to all the items. We hope the manuscript after careful revisions meet your high standards. Below we provide the point-by-point responses. All modifications in the manuscript have been highlighted as tracked changes.

**Editor Comments**

The reviewers appreciated your attention to their suggestions and had only a few  minor comments. Please note the request to include a statement about your missing data within the methods section of your manuscript. We would encourage you to resubmit these minor corrections as soon as you can.

***Author response:*** We thank the Editor for the feedback. We have now added a statement about the missing data within the methods section of the manuscript and have also addressed the other feedback raised by the reviewers as below.

**Reviewer #1**

The authors have addressed the comments I made earlier. I have a couple of minor additional suggestions for them to consider.

1. Intro: One minor suggestion to update this sentence by inserting something where I put in caps: "By uncovering the specific contextual, demographic, and psychosocial factors [ASSOCIATED WITH ...], this study provides insights into the mechanisms that contribute to the effectiveness of the intervention across diverse populations."

***Author response:*** We thank the reviewer for the feedback. We have now revised the sentence as suggested to improve clarity (See Page 6, Lines 109-112).

***Modifications:*** *“By uncovering the specific contextual, demographic, and psychosocial factors associated with differential engagement with the WMG-E tool and variation in the identification and support of unmet social care needs, this study provides insights into the mechanisms that contribute to the effectiveness of the intervention across diverse populations.”*

2. Discussion

In discussion line 298, the authors introduce the term "diagnostic overload". They cite Garg paper, which does talk about how lack of effective intervention can increase inequity. But I don't think it's appropriate to quote "diagnostic overload" here. It doesn't come from Garg paper--I went back to read that paper out of curiosity. Garg's paper refers to the inadequacy of interventions, not diagnostic overload. I think these are subtly different and I would distinguish between them.

***Author response:*** We agree with the reviewer and we have revised the text to more accurately reflect the argument presented in Garg’s work, namely that screening for social needs in the absence of effective, accessible, and timely intervention pathways may inadvertently exacerbate inequities (See Page 20, Lines 303-312).

***Modifications:*** *“The increase in reported social care needs among Class 2 participants likely reflects the identification of unmet needs without sufficient capacity or intensity of intervention to address them, a phenomenon highlighted in prior work examining social risk screening in healthcare settings [26]. In such contexts, screening without effective, accessible, and timely intervention pathways may unintentionally exacerbate inequities, particularly for families facing structural and systemic barriers to care, including limited financial resources, reduced local service availability, transportation challenges, and difficulties balancing work or caregiving responsibilities.”*

3. Also one very minor grammatical thing I noticed in this sentence below. I think it is missing a dash where I put in brackets: "In contrast, Class 2 – representing the most disadvantaged group [—] reported higher unmet social care needs and a persistent trend of increasing needs post-intervention, indicating that more intensive support might be required beyond the digital intervention alone.

***Author response:*** We have now added the dash as suggested (See Page 19, Lines 274-277).

***Modifications:*** *“In contrast, Class 2 – representing the most disadvantaged group – reported higher unmet social care needs and a persistent trend of increasing needs post-intervention, indicating that more intensive support might be required beyond the digital intervention alone.”*

**Reviewer #2**

1. Overall satisfied with the responses and the resulting changes to the paper. About my comment on missing data, the response I received needs to be included in the methods section.

***Author response:*** We thank the reviewer for the feedback. We have added the sentence about missing data in the data analysis section (See Pages 8-9, Lines 166-168).

***Modifications:*** *“All data were complete except for the ‘current service use’ variable, which had three missing cases. Given the minimal extent of missing data, multiple imputation was not undertaken, and analyses were conducted using available-case data.”*
